# Enhanced cognitive behaviour therapy (CBT-E) for severe and extreme anorexia nervosa in an outpatient eating disorder unit at a public hospital: a quality-assessment study

**DOI:** 10.1186/s40337-021-00499-1

**Published:** 2021-11-02

**Authors:** Stein Frostad, Simona Calugi, Caroline B. N. Engen, Riccardo Dalle Grave

**Affiliations:** 1 Division of Psychiatry, Haukeland University Hospital, Bergen, Norway; 2 Department of Eating and Weight Disorders, Villa Garda Hospital, Garda, VR Italy

**Keywords:** Severe and extreme anorexia nervosa, Adults, Cognitive behaviour therapy, Outpatient

## Abstract

**Background:**

The aim of this quality-assessment study was to determine the outcome of patients with severe and extreme anorexia nervosa (AN) in a real-world outpatient setting.

**Methods:**

Twenty-one adults with AN and a body mass index (BMI) of < 16 were recruited from consecutive referrals to an outpatient clinic at a public hospital in Western Norway. All enrolled patients were provided with enhanced cognitive behaviour therapy (CBT-E) to treat their AN, commencing between January 2013 and December 2016. Their BMI was recorded at baseline, at the end of CBT-E and 1 year after the end of treatment.

**Results:**

Ten patients completed the CBT-E treatment and achieved a large weight gain with the change remaining stable at follow-up. Eleven patients did not complete the treatment but had a significant increase in BMI at the premature end of treatment. One year after end of therapy 14/21 (66.7%) of the patients had BMI above 18.5 kg/m^2^. No severe complications were observed during therapy.

**Conclusions:**

Although 52.4% of the patients did not complete outpatient CBT-E, the findings of this quality-assessment study support previous findings indicating that CBT-E may represent a valid alternative to inpatient treatment in patients with severe and extreme AN.

## Introduction

Patients with severe (BMI 15–15.99) or extreme (BMI < 15) AN [1] are often managed in an inpatient setting. However, some studies indicate that CBT-E can be suitable for these patients, provided that their medical conditions is stable [2]. The aim of this quality-assessment study was to determine the outcome of patients with severe and extreme AN in a real-world outpatient setting,

## Methods

This quality-assessment study was performed at the department of eating disorders (DED) of the Psychiatric Clinic at Haukeland University Hospital, Bergen, Western Norway. The DED is a tertiary specialist ED unit that forms part of the public healthcare system in Norway.

**Fig. 1:**
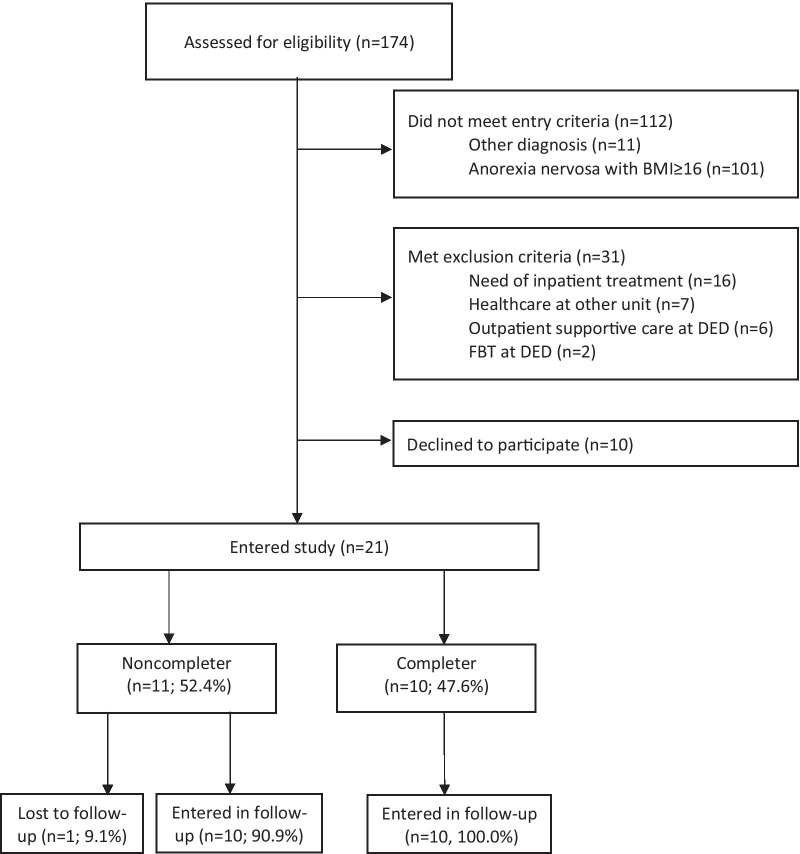
CONSORT diagram. *BMI* body mass index, *CBT-E* enhanced cognitive behaviour therapy for eating disorders, *FBT* family-based treatment, *AN* anorexia nervosa, *DED* department of eating disorders. Exclusion criteria: need of inpatient treatment at DED, healthcare was delivered at other unit, outpatient supportive care at DED because unable to perform psychotherapy or FBT at DED. Declined to participate; was offered outpatient CBT-E but declined to start (n = 9), other reason (n = 1)

The study sample comprised 21 consecutive patients aged between 17 and 51 years with severe or extreme AN (i.e. BMI < 16 kg/m^2^) enrolled between 1 January 2013 and 31 December 2016. Details of the patient flow to the DED are published elsewhere [3]. Figure 1 shows the recruitment and retention data for patients.

EDs were diagnosed based on a clinical evaluation according to DSM-5 criteria [1]. BMI < 18.5, 15.0–15.99, and < 15.0 were applied as the inclusion criteria for AN, severe AN and extreme AN, respectively**.** The Mini International Neuropsychiatric Interview (version 6.0) [4] was used to screen for comorbid psychiatric disorders at baseline. At baseline the patients also completed the Norwegian versions of the Eating Disorder Examination Questionnaire version 6.0 [5] and the Clinical Impairment Assessment Questionnaire [6].

A physician assessed the patients before they received healthcare at the DED. Healthcare was overseen by an experienced medical specialist (S.F.).

CBT-E was performed as described in detail in the complete treatment manual [5] and in previous publications on DED treatment [3]. The patients did not receive any other ED psychotherapy while they were receiving CBT-E.

### Statistics

All statistical analyses were conducted using SPSS statistical package version 26.0 and R version 3.6.0 [7]. Wilcoxon rank sum test was conducted to compare the BMI at baseline, at the end of treatment (EOT) and at 1 year after the EOT. Cohen’s *f* effect sizes for within-sample changes in BMI from baseline to end of treatment were calculated, with values of above 0.4 representing large effect size [8].

## Results

The sociodemographic background and ED characteristics of the enrolled patients are presented in Table 1. The mean baseline BMI of the overall cohort was 14.8.

**Table 1:** Characteristics of 21 consecutive patients starting enhanced cognitive behaviour therapy (CBT-E) for anorexia nervosa

Characteristics	All patients	Completers	Non-completers	*p* Value*
Age, years, mean (SD)	25.5 (7.9)	25.9 (4.2)	24.3 (10.3)	0.115
Mean age at eating disorder onset, years, mean (SD)	17.7 (6.0)	18.1 (5.0)	17.5 (7.5)	0.357
Duration of illness, years, mean (SD)	7.3 (6.6)	7.8 (6.2)	6.8 (6.6)	0.905
Binge-eating purge type, n (%)	5 (23.8%)	2 (20.0%)	3 (27.0%)	1.000
Restricting type, n (%)	16 (76.2%)	8 (80.0%)	8 (73.0%)	1.000
One or more axis-1 disorders, n (%)	14 (66.7%)	7 (70.0%)	7 (64.0%)	1.000
*Most-frequent other axis-1 disorders, n (%)*				
Depression	9 (42.9%)	6 (60.0%)	3 (27.0%)	0.198
Post-traumatic stress disorders	3 (14.3%)	0	3 (27.0%)	0.214
Anxiety disorder	1 (5.0%)	1 (10.0%)	0	0.476
Obsessive–compulsive disorder	1 (5.0%)	0	1 (9.7%)	1.000
Using psychopharmacological treatment	5 (23.8%)	4 (40.0%)	1 (9.0%)	0.149
Using laxatives	5 (28.6%)	2 (20.0%)	3 (27.0%)	1.000
Excessive exercise	5 (23.8%)	2 (20.0%)	3 (27.0%)	1.000
Previous treatment of eating disorder	16 (72.6%)	7 (70.0%)	9 (82.0%)	0.635
Previous inpatient treatment	10 (47.6%)	4 (40.0%)	6 (55.0%)	0.670
*Living situation, n (%)*				
Single	15 (71.4%)	7 (70.0%)	8 (73.0%)	1.000
Married or living with a partner	4 (19.0%)	2 (20.0%)	2 (18.0%)	1.000
Separated or divorced	2 (9.5%)	1 (10.0%)	1 (9.0%)	1.000
*Occupation*				
Student	12 (57.0%)	5 (50.0%)	7 (64.0%)	0.670
Employee	8 (38.0%)	5 (50.0%)	3 (27.0%)	0.387
Unemployed	1 (5.0%)	0	1 (9.0%)	1.000
*Severity*				
Body mass index, kg/m^2^, mean (SD)	14.8 (1.1)	14.9 (1.2)	14.6 (1.1)	0.503
Severe anorexia nervosa, n (%)	11 (52.4%)	6 (60.0%)	5 (45.5%)	0.670
Extreme anorexia nervosa, n (%)	10 (47.6%)	4 (40.0%)	6 (54.5%)	0.670
Global EDE-Q score (11/21 patients), mean (SD)	4.0 (0.9)	3.9 (0.4) (n = 5/10)	4.1 (1.2) (n = 6/11)	0.714
CIA score (11/21 patients), mean (SD)	37.2 (9.8)	37.3 (8.6)	37.0 (11.5)	0.792

*EDE-Q* eating disorder examination questionnaire version 6.0, *CIA* clinical impairment assessment questionnaire. *Statistical tests include Wilcoxon rank sum test or Fisher exact test, as appropriate

Nearly half of the 21 patients who started the treatment (*n* = 10, 47.6%) completed it (completers); the remaining 11 (52.4%) ended treatment prematurely (non-completers). Completers and non-completers were similar in age, BMI, number of years with an ED, rate of psychiatric comorbidity, number of previous ED treatment attempts and living situation (as listed in Table 1).

### Outcomes among completers

The mean BMI values at baseline, EOT and 1 year after the EOT for the ten completers are shown in Table 2. There was a significant weight gain at EOT (Wilcoxon rank sum test: *p* = 0.0019), and the effect size for this change was large (Cohen’s *f* = 0.63).

**Table 2:** BMI of completers and non-completers

	Completers	SD	*p*	Cohen’s *f*	Non-completers	SD	*p*	Cohen’s *f*
Start of treatment	14.9	1.2			14.6	1.1		
End of treatment	19.4	1.2	0.002	0.63	16.5	2.5	0.019	0.51
Follow-up	19.2	1.6	0.594		18.0	3.4	0.575	

BMI, mean and standard deviation (SD), at start of treatment, end of treatment and follow-up. Wilcoxon rank sum test was used for comparison of BMI at start of treatment with BMI at end of treatment and for comparison of BMI at end of treatment with BMI at follow-up, *p* < 0.05 was regarded as statistical significant. Cohen’s *f* > 0.4 was regarded as a large effect

The percentage of completers achieving BMI ≥ 18.5 was 80% at EOT. The mean duration of CBT-E was 14.6 (SD 8.2) months (median, 13.2 months), and the mean number of sessions was 60.9 (SD 28.5) (median, 66.0).

Weight data 1 year after the EOT was available for all completers. There was no significant difference between BMI at EOT and BMI 1 year after the EOT for completers (Wilcoxon rank sum test: *p* = 0.59). At the 1-year follow-up, 80% of completers had BMI ≥ 18.5.

None of the completers entered inpatient care for ED in the first year after CBT-E.

### Outcomes among non-completers

The BMI also increased significantly among the 11 non-completers as shown in Table 2. The percentage of non-completers presenting with BMI ≥ 18.5 at the last CBT-E session was 27% (3 of 11 patients). Follow-up took place at DED or in collaboration with DED (*n* = 5) or at district psychiatric centres in the Bergen area (*n* = 5). One of the non-completers was lost to follow-up. One year after premature end of CBT-E, the non-completers had a mean BMI of 18.01 (SD 3.4) (median, 19.15 kg/m^2^) and six of the eleven non-completers (55%) achieved BMI  ≥ 18.5 one year after premature end of treatment.

Thus, among the 21 patients with severe or extreme anorexia nervosa who started outpatient CBT-E 14 (66.7%) had BMI ≥ 18.5 at follow-up.

## Discussion

The present findings support previous studies indicating that CBT-E may be suitable for severe and extreme AN patients without acute medical complications. 20/21 (95%) of the patients who started outpatient CBT-E received follow-up healthcare at DED or at their local district psychiatric center. Among these 20 patients 14 (70%) had BMI > 18.5 kg/m^2^ one year after EOT. This quality-assessment study also supports previous findings indicating that BMI alone has limited value as a criterion for inpatient care. Further studies with larger samples are needed to evaluate the moderating effects of BMI and who is more likely not to complete treatment [9], and may need other forms of treatment [10].

## Data Availability

Authors will make de-identified data available upon reasonable request.

## Abbreviations

AN: Anorexia nervosa
BMI: Body mass index
CBT-E: Enhanced cognitive behaviour therapy
DED: Department of eating disorders
DSM-5: Diagnostic and statistical manual of mental disorders, fifth edition
ED: Eating disorder
EOT: End of treatment
FBT: Family-based treatment
IBM SPSS: International business machines statistical package for the social sciences
NOK: Norwegian krone
REK: Regional ethical committee
